# Gonad Transcriptome Analysis of High-Temperature-Treated Females and High-Temperature-Induced Sex-Reversed Neomales in Nile Tilapia

**DOI:** 10.3390/ijms19030689

**Published:** 2018-02-28

**Authors:** Li Xue Sun, Jian Teng, Yan Zhao, Ning Li, Hui Wang, Xiang Shan Ji

**Affiliations:** Shandong Provincial Key Laboratory of Animal Biotechnology and Disease Control and Prevention, Shandong Agricultural University, 61 Daizong Street, Taian 271018, China; luckyfish.sun@gmail.com (L.X.S.); tengjiannc@163.com (J.T.); yzhao@sdau.edu.cn (Y.Z.); aitienan@163.com (N.L.); wanghui2328@sdau.edu.cn (H.W.)

**Keywords:** high temperature, masculinization, transcriptome, Nile tilapia, lasting effect

## Abstract

Background: Nowadays, the molecular mechanisms governing TSD (temperature-dependent sex determination) or GSD + TE (genotypic sex determination + temperature effects) remain a mystery in fish. Methods: We developed three all-female families of Nile tilapia (*Oreochromis niloticus*), and the family with the highest male ratio after high-temperature treatment was used for transcriptome analysis. Results: First, gonadal histology analysis indicated that the histological morphology of control females (CF) was not significantly different from that of high-temperature-treated females (TF) at various development stages. However, the high-temperature treatment caused a lag of spermatogenesis in high-temperature-induced neomales (IM). Next, we sequenced the transcriptome of CF, TF, and IM Nile tilapia. 79, 11,117, and 11,000 differentially expressed genes (DEGs) were detected in the CF–TF, CF–IM, and TF–IM comparisons, respectively, and 44 DEGs showed identical expression changes in the CF–TF and CF–IM comparisons. Principal component analysis (PCA) indicated that three individuals in CF and three individuals in TF formed a cluster, and three individuals in IM formed a distinct cluster, which confirmed that the gonad transcriptome profile of TF was similar to that of CF and different from that of IM. Finally, six sex-related genes were validated by qRT-PCR. Conclusions: This study identifies a number of genes that may be involved in GSD + TE, which will be useful for investigating the molecular mechanisms of TSD or GSD + TE in fish.

## 1. Introduction

Nile tilapia (*Oreochromis niloticus*) are the third most important aquaculture fish after carp and salmon, and males grow significantly faster than females. The whole genome of Nile tilapia was sequenced in 2012, and genome assemblies have been released in the National Center for Biotechnology Information (NCBI) [1,2]. A considerable amount of previous research has centered on the sex determination mechanism and all-male production in Nile tilapia, making this species an ideal one for studying molecular signaling cascades and gene expression networks during sex determination and differentiation [1,3].

According to the role of temperature in sex determination, the sex-determining mechanisms in fish can be broadly classified as genotypic (GSD), temperature-dependent (TSD), or genotypic plus temperature effects (GSD + TE) [4]. The first evidence of TSD in fish species was obtained in the Atlantic silverside, *Menidia menidia* [5]. Fish species with TSD exhibit a sex ratio response to temperature within the range of temperature during development in the wild and have no sex chromosomes [4]. Although there are sex chromosomes in many fish, artificially high or low temperatures during critical thermosensitive periods (TSP) also result in their sex ratio changes. These fish are classified as GSD + TE. For example, Nile tilapia has a male heterogametic (XX/XY) sex determination system [6,7,8], but high-temperature treatment applied approximately 10 days post-fertilization and lasting from 10 to 28 days in Nile tilapia can induce XX genotypic female sex reversal to XX neomales [9,10,11,12,13]. For GSD + TE fish, high or low temperatures can override original genetic influences and switch on new sex differentiation pathways when the gonad is undifferentiated [5,10,13]. Thus, the Nile tilapia is a perfectly suited model and indeed one of the best documented fish species in terms of sex ratio shifts in response to temperature. 

The effects of temperature on gene expression during sex differentiation have been investigated in different teleost species, including the African catfish (*Clarias gariepinus*) [14], European sea bass (*Dicentrarchus labrax*) [15,16], Japanese flounder (*Paralichthys olivaceus*) [17], pejerrey (*Odontesthes bonariensis*) [18], and Nile tilapia [19,20]. Many studies have shown that high-temperature treatment affects mRNA expression of many genes such as *dmrt1*, *amh*, *sox9a*, *cyp19a1*, *foxl2*, *wt1*, *sf1*, and *hsps* [19,20,21,22]. Furthermore, sex steroids have long been suspected to play a role in TSD and GSD + TE, as these molecules are known to play a critical role in sex differentiation in fish species [22,23,24]. If the expression of *cyp19a1a* or other steroidogenic genes is affected during high-temperature treatment, sex steroid levels in the gonad or serum are altered [25,26]. Therefore, the mRNA expression changes of related genes during high- or low-temperature treatment are an important cue for fish sex reversal in TSD or GSD + TE. How the developing gonad translates temperature cues into specific biochemical signals remains unanswered. There is a crucial need to gain a comprehensive picture in order to fully understand the molecular mechanisms underlying TSD or GSD + TE during high- or low-temperature treatment.

In this study, XX sex reversal neomales were crossed with XX females, and three all-female families of Nile tilapia were developed. RNA-Seq analyses of the gonads from control females (CF), high-temperature-treated females (TF), and high-temperature-induced neomales (IM) from a single family were performed. The objectives of the present study were: (1) to compare the histological and transcriptomic differences between CF and TF gonads and provide a comprehensive insight into the long-lasting effects of high-temperature treatment on the transcriptome and gonad development in Nile tilapia; (2) to compare the histological and transcriptomic differences of CF, TF, and IM gonads and reveal the mechanisms underlying high-temperature-induced partial female sex reversal to neomales; (3) to identify high-temperature treatment-related differentially expressed genes (DEGs).

## 2. Results

### 2.1. High-Temperature Treatment Influenced the Sex Ratio

As shown in Figure S1, the neomales used for the development of all-female families were identified by using PCR-based genetic sex identification. In this study, three all-female families were developed by crossing identified XX neomales with XX females. When the larvae in three all-female families were subjected to 36 °C high-temperature treatment from five to 17 dph (days post-hatching), the proportions of males were 74% (family 1), 86% (family 2), and 90.4% (family 3) after treatment. Consistent with previous reports [9], our results also showed that the high-temperature treatment induced a change in sex ratio towards males and that the sex reversal ratio was family-specific. These results further verified the existence of a clear genotype-by-environment (GxE) interaction during the sex determination and differentiation processes in Nile tilapia. The remaining females continued to develop as females despite the high-temperature treatment. We decided to investigate whether the female transcriptome changed after high-temperature treatment.

### 2.2. Effects of High-Temperature Treatment on Gonadal Development

**Figure 1:**
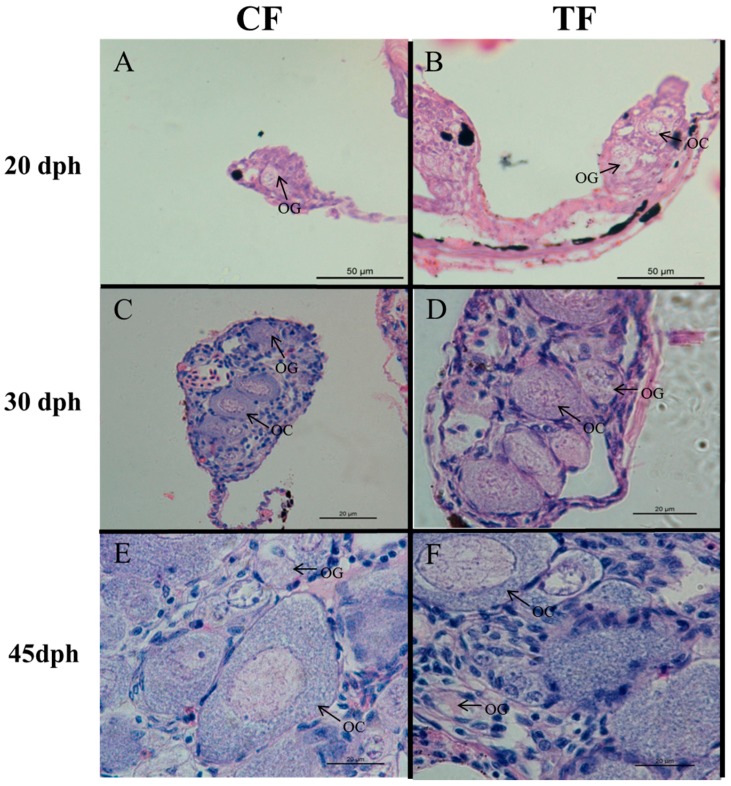
Gonad histology of control females (CF) and high-temperature-treated females (TF) at various developmental stages. (**A**,**C**,**E**) gonad of 20, 30 and 45 dph control female, respectively; (**B**,**D**,**F**): gonad of 20, 30 and 45 dph high-temperature-induced males, respectively OG: oogonia; OC: oocyte; dph: days post-hatching.

**Figure 2:**
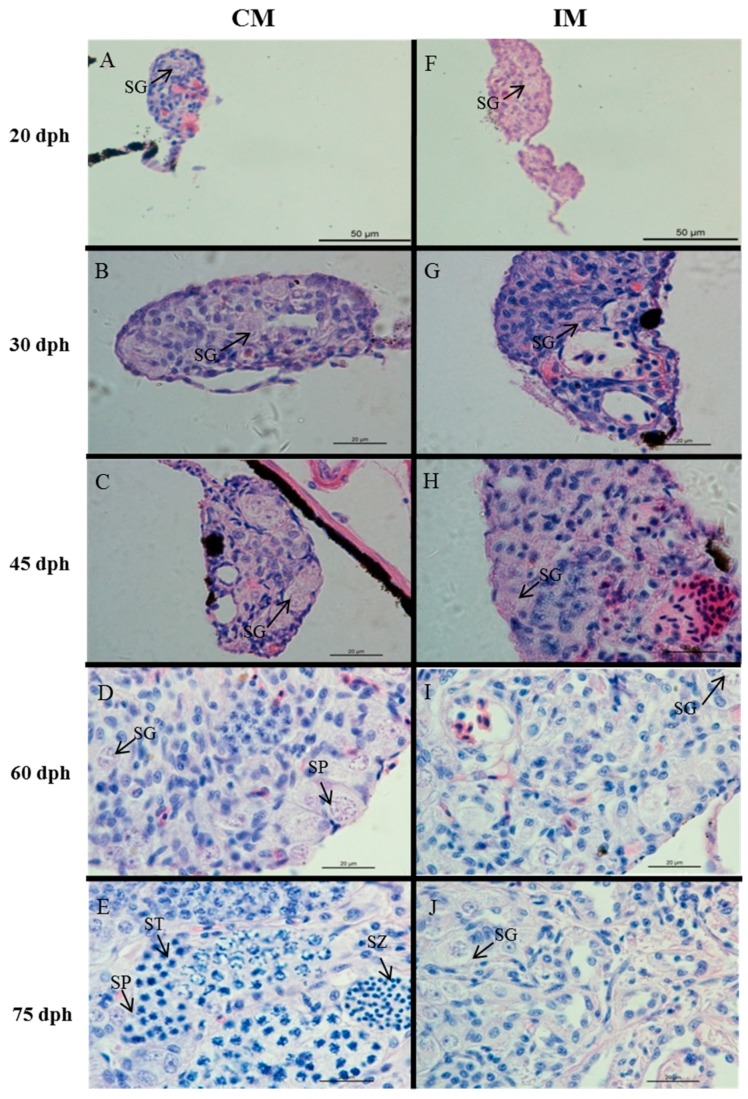
Gonad histology of control males (CM) and high-temperature-induced males (IM) at various developmental stages. (**A**–**E**): gonad of 20–75 dph control males; (**F**–**J**): gonad of 20–75 dph high-temperature-induced males; SG: spermatogonia; SP: spermatocyte; ST: spermatid; SZ: spermatozoa; dph: days post-hatching.

The juveniles CF, TF, and IM from all-female family 3 and CM (control males) from the other control family at various stages were used for gonadal histology. At 20 dph, we observed small and rounded oogonia with a spherical nucleus occupying almost the entire cell in CF and TF (Figure 1). At 30 dph, larger oocytes were found in CF and TF. At 45 dph, there was still no significant difference in the gonadal histological morphology between CF and TF, and oocytes became much larger, round, or polygonal and had a large oval nucleus in the center. At 20, 30, and 45 dph, there were no significant differences of gonad morphology between CM and IM (Figure 2). At 60 dph, neomales possessed relatively fewer spermatogonia compared with CM. At 75 dph, germ cells of different stages were found in CM, including spermatogonia, spermatocytes, and spermatids. However, most germ cells remained at the spermatogonial stage in IM. At six months, the gonad in IM matured, and milky milt could be squeezed from some of the IM fish. Our previous study also reported that there were no significant differences between CM and IM in the number of spermatocytes and spermatozoa at maturity [27].

### 2.3. Illumina Sequencing and Sequence Assembly

According to previous reports, the onset time point of ovarian maturation of Nile tilapia reared at 28 °C was 115 dph [28]. In order to investigate the lasting effect of high-temperature treatment on gonad development, the fish at 115 dph were sacrificed for sampling. The mean body weight of three females in the control group (CF), three females in the high-temperature-treated group (TF), and three neomales in the high-temperature-induced group (IM) in family 3 used for RNA-Seq and qRT-PCR analysis was, respectively, 102.12 ± 4.56 g, 102.58 ± 3.69 g, and 102.86 ± 4.36 g, which allowed us to ignore the effects of growth on gene expression. We performed RNA-seq analysis on these randomly selected nine samples, which yielded a total of nine cDNA libraries. A total of 149 million raw reads and 124 million clean reads were generated from the samples (Table S1). Of all the clean reads, an average of 80.09% of the reads was mapped to the high-quality Nile tilapia reference genome, which is composed of 5677 scaffolds with an N50 scaffold size of 26.68 Mbp [8]. After aligning with the Nile tilapia genome, 29,072, 29,235, and 29,675 genes were obtained from CF, TF, and IM, respectively.

### 2.4. Identification of Differentially Expressed Genes (DEGs)

**Figure 3:**
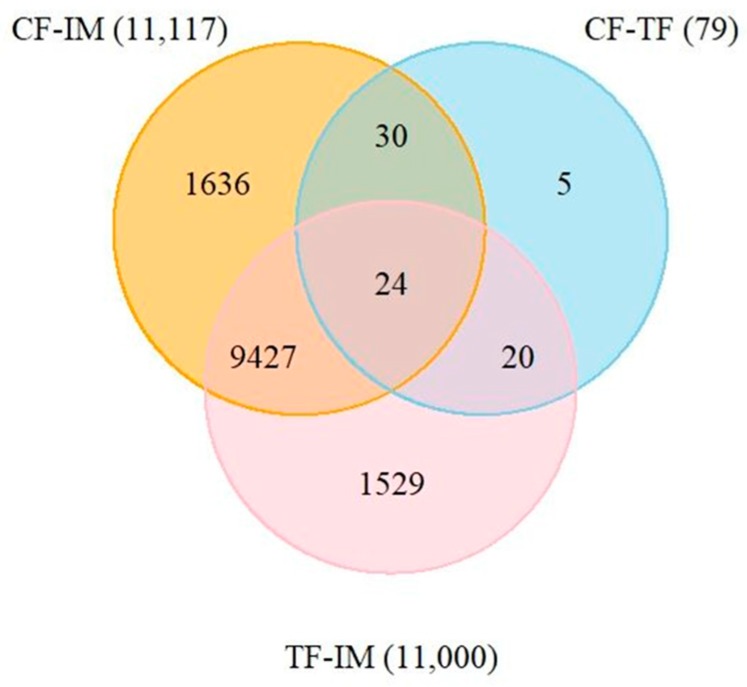
Analysis of DEGs by Venn diagram showing the number of DEGs in CF–TF, CF–IM, and TF–IM comparisons. All DEGs were determined on the basis of the statistical significance (false discovery rate (FDR)  < 0.05).

To evaluate the differential expression of genes between the control group and the high-temperature-treated group, multiple comparisons were conducted between CF and TF, CF and IM, and TF and IM. DEseq was used to screen differentially expressed genes (DEGs) with a false discovery rate (FDR) adjusted *p*-value ≤ 0.05. The results revealed extensive gene expression changes during the course of the high-temperature treatment. Overall, the comparison of CF and TF showed 79 DEGs, with 34 upregulated and 45 downregulated genes (Table 1). When comparing CF and IM, 11,117 DEGs were identified, of which 5779 were upregulated and 5338 were downregulated. In the comparison between TF/ and IM, 11,000 DEGs were identified, of which 5607 were upregulated and 5393 were downregulated. Detailed information of these DEGs is shown in Table S2. These DEGs were searched against the NCBI non-redundant (NR), Swiss-Prot, KEGG, GO, and COG databases by using BLASTX with a cut-off *E*-value of 1 × 10^−5^. In total, 669,589 and 9490 DEGs were annotated, representing 83.5%, 86.3%, and 86.3% of total DEGs in the comparisons of CF with TF (79), CF with IM (11,117), and TF with IM (11,000), respectively (Figure S2). Venn diagram analysis showed that among the 79 DEGs in the CF–TF comparison, 54 were also included in the list of DEGs of the CF–IM comparison, and 44 in the list of DEGs of the TF–IM comparison (Figure 3). Among the 11,000 DEGs in the TF–IM comparison, 9471 DEGs were also included in the list of DEGs of the CF–IM comparison. Previously, XX/XY sex determination systems have been described on both linkage group 1 (LG1) and linkage group 23 (LG23) in Nile tilapia [6,7,8]. The physical locations of the identified DEGs was analyzed. Four and two DEGs in the CF–TF comparison were found to be located on LG1 and LG23, with no enrichments for DEGs on LG1 or LG23 (Table S3). However, the DEG *cyp19a1a*, an important candidate gene in TSD and GSD + TE, was located on LG1. Also, no enrichments for DEGs in the CF–IM or TF–IM comparisons were identified on LG1 or LG23. Additionally, 373 and 314 DEGs in the CF–IM comparison were respectively located on LG1 and LG23. Similarly, 369 and 314 DEGs in the TF–IM comparison were respectively located on LG1 and LG23. 

### 2.5. GO and KEGG (Kyoto Encyclopedia of Genes and Genomes) Enrichment Analysis of DEGs

GO enrichment analysis was performed to investigate the possible roles of these DEGs. Of the three main categories (cellular components, molecular functions, and biological processes), there were 33, 4041 and 3957 DEGs in the CF–TF, CF–IM, and TF–IM comparisons, respectively, for which GO annotations related to molecular functions were available (Figures S3–S5). This suggested that a series of molecular events occur in Nile tilapia gonads after high-temperature treatment. Some GO terms that were enriched in the downregulated DEGs of the CF–IM but not the CF–TF comparison, were related to female-specific pathways, such as female gonad development (GO: 0008585) and female meiosis I (GO: 0007144). The downregulated DEGs of the CF–IM comparison were also enriched with GO terms related to male-specific pathways, such as spermatid development (GO: 0007286) and spermatogenesis (GO: 0007283). However, a GO term related to the male pathway sperm capacitation (GO: 0048240) was enriched in the upregulated DEGs of the CF–IM comparison. These results suggested that the high-temperature treatment in the early gonad differentiation period had an effect on the gonad development of IM. Detailed information of GO enrichment analysis for DEGs in three comparisons is shown in Figures S3–S5.

KEGG enrichment analysis was also performed for DEGs identified in the three comparisons according to the KEGG database. Pathways with a *p*-value ≤ 0.05 were significantly enriched. A total of 59, 263 and 261 KEGG pathways were differentially expressed in the CF–TF, CF–IM, and TF–IM comparisons (Table S4). Many significantly enriched KEGG pathways involved in sex determination and gonad development, such as ovarian steroidogenesis, steroid biosynthesis, steroid hormone biosynthesis, PPAR signaling pathway, MAPK signaling pathway, GnRH signaling pathway, PI3K-Akt signaling pathway, estrogen signaling pathway, progesterone-mediated oocyte maturation, and oocyte meiosis were identified in the CF–IM and TF–IM comparisons. In the CF–TF comparison, many significantly enriched KEGG pathways were also involved in sex determination and gonad development, such as ovarian steroidogenesis, steroid hormone biosynthesis, GnRH signaling pathway, MAPK signaling pathway, and PI3K-Akt signaling pathway. Detailed information of the KEGG pathways in the three comparisons is shown in Table S4.

### 2.6. DEGs in the CF–TF comparison

**Figure 4:**
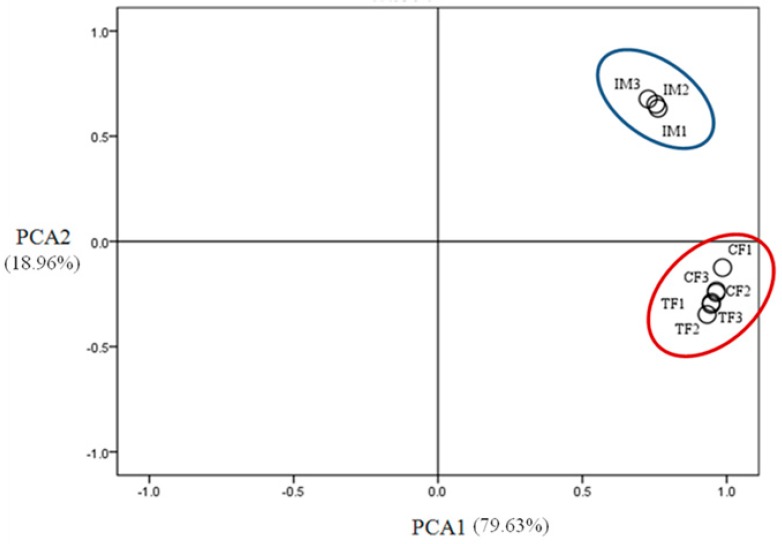
Clustering of control females (CF), high-temperature-treated females (TF), and high-temperature-induced neomales (IM) based on their gonadal transcriptome profiles by principal component analysis (PCA). Three individuals in CF and three individuals in TF formed a cluster (red circle), and three individuals in IM formed a distinct cluster (blue circle) on a PCA plot.

Although histological observations of the gonads showed that there were no significant differences between CF and TF at various development stages (Figure 1), 79 DEGs were found in the CF–TF comparison (Table S2). For instance, the expression level of *cyp19a1a*, which is critical for estrogen synthesis in Nile tilapia [29], was significantly downregulated after high-temperature treatment. Other downregulated DEGs included G protein-coupled receptor 6-like, mitogen-activated protein kinase kinase kinase 3-like isoform X5, etc. However, the expression levels of many DEGs were upregulated, such as that of forkhead box protein O4-like isoform X1, nuclear receptor-binding protein, cell division control protein 42 homolog isoform X1, etc. These results indicated that the high-temperature treatment at early sex differentiation stages had long-lasting effects on the expression level of some DEGs, though it did not induce sex reversal of these females. Consistent with the number of DEGs identified in the multiple comparisons between CF and TF, principal component analysis (PCA) indicated that three individuals in CF and three individuals in TF formed a cluster (Figure 4). This showed that TF had similar gonadal expression profiles to CF.

### 2.7. DEGs in the CF–IM comparison

**Figure 5:**
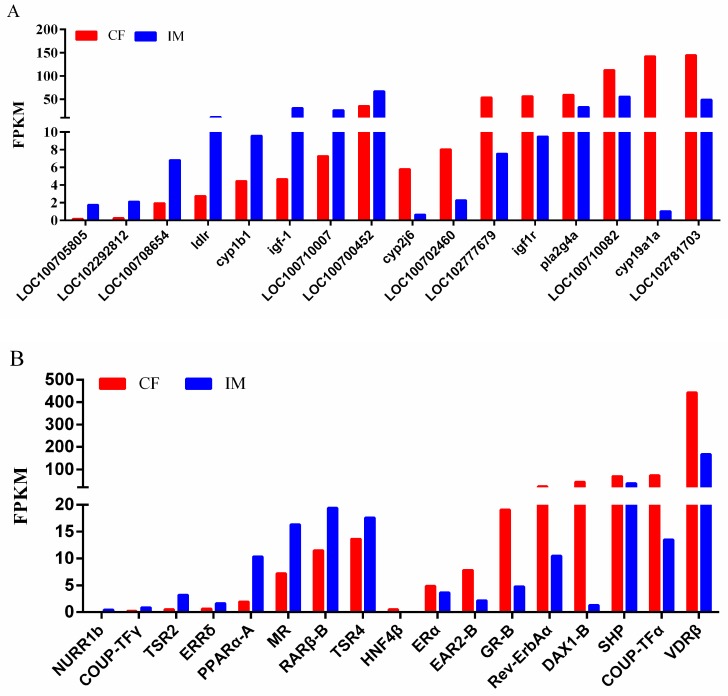
Expression profiles of major genes involved in steroid biosynthesis and metabolism (**A**) and from the nuclear receptor family (**B**) in Nile tilapia, displayed on the basis of their FPKM values from RNA-seq data from the CF–IM comparison. The FDR values of all comparisons are <0.05.

In the CF–IM comparison, we found 11,117 DEGs, and PCA indicated that three individuals in CF and three individuals in IM formed a distinct cluster on the PCA plot (Figure 4). This showed that IM had a gonadal expression profile distinct from that of CF. Sex steroids and nuclear receptors sensing steroids play important roles in fish sex determination and sex differentiation and are particularly involved in female sex reversal to males after high-temperature treatment [21,30]. Therefore, the expression patterns of steroidogenic-related DEGs in the CF–IM comparison were examined in this study (Figure 5A). Upregulated steroidogenic-related genes in IM compared with CF included 3-keto-steroid reductase (LOC100708654), adenylate cyclase type 8 (LOC100705805), adenylate cyclase type 1-like (LOC102292812), cytochrome P450 1B1 (*cyp1b1*), low-density lipoprotein receptor (*ldlr*), insulin-like growth factor I (*igf-1*), adenylate cyclase type 3 (LOC100710007), and cAMP-dependent protein kinase catalytic subunit alpha (LOC100700452) (Figure 5A). However, cytochrome P450 2J6 (*cyp2j6*), adenylate cyclase type 6 (LOC100702460), insulin receptor-like (LOC102777679), insulin-like growth factor 1 receptor-like (*igf-1r*), cytosolic phospholipase A2 (*pla2g4a*), guanine nucleotide-binding protein G(s) subunit alpha (LOC100710082), and cytochrome P450 aromatase (*cyp19a1a*) were found to be significantly downregulated in IM compared with CF. Of these DEGs, the expression of 3-keto-steroid reductase (LOC100708654) and cytochrome P450 1B1 (*cyp1b1*), which are involved in androgen biosynthesis and estrogen metabolism, respectively, were significantly upregulated in IM compared with CF. However, *cyp19a1a* and guanine nucleotide-binding protein G(s) subunit alpha isoforms short (LOC100710082), which are involved in estrogen biosynthesis, showed significant downregulation in IM compared with CF. These observations revealed that the high-temperature treatment directly or indirectly downregulated the mRNA expression of estrogen biosynthesis-related genes and upregulated the mRNA expression of androgen biosynthesis-related genes.

We then identified seventeen differentially expressed nuclear receptors (NR) in the CF–IM comparison, considering the previously identified NR from Nile tilapia [30]. Eight genes, namely, *TSR2* (*NR2C1*), *TSR4* (*NR2C2*), *ERRδ* (*NR3B4*), etc., were significantly upregulated in IM compared with CF (Figure 5B; Table S5). However, nine genes, namely, *ERα* (*NR3A1*), *DAX1-B* (*NR0B1B*), *ERRδ* (*NR3B4*), *SHP* (*NR0B2A*), *GR-B* (*NR3C1B*), etc., were significantly downregulated in IM compared with CF. Among them, *ERα* and *ERRδ* are nuclear receptor sensing steroids.

### 2.8. Identification of DEGs Shared in the CF–TF and CF–IM comparisons

In order to find the DEGs in the CF–TF comparison involved in sex differentiation and sex determination, we performed a Venn diagram analysis that showed that the two comparison groups (CF–TF and CF–IM) shared 54 DEGs. Among the 54 DEGs, 45 were annotated genes and nine were not annotated in the NCBI non-redundant (nr) database (Table 2). These shared DEGs were subjected to further analysis to investigate common events after high-temperature treatment. Most of the 54 DEGs (44 DEGs, accounting for 81.5% of total shared DEGs) showed changes to a similar direction in the two comparisons, and 10 DEGs showed different expression changes between in the CF–TF and CF–IM comparisons, suggesting that sex-reversed neomales altered the expression patterns of the 10 genes during gonad development (Table 2 and Table S2). Although the phenotypical sex in TF did not change, 44 DEGs were also significantly downregulated or upregulated in TF compared with CF, and many of them possibly did not reach the threshold resulting in female sex reversal into neomales, as Navarro-Martín et al. reported for cyp19a1a [16]. Many of the 44 DEGs were found in many sex differentiation-related KEGG pathways, such as steroid hormone biosynthesis (ko00140), axon guidance (ko04360), MAPK signaling pathway (ko04010), Ras signaling pathway (ko04014), Fc gamma R-mediated phagocytosis (ko04666), etc. (Table S4). Therefore, these 44 DEGs appeared as important candidate genes for investigating the molecular mechanisms for fish GSD + TE or TSD. Among the 44 DEGs, 27 DEGs were significantly downregulated in the two comparisons, and 17 were upregulated (Table 2 and Table S2).

### 2.9. Validation of DEGs by qRT-PCR

**Figure 6:**
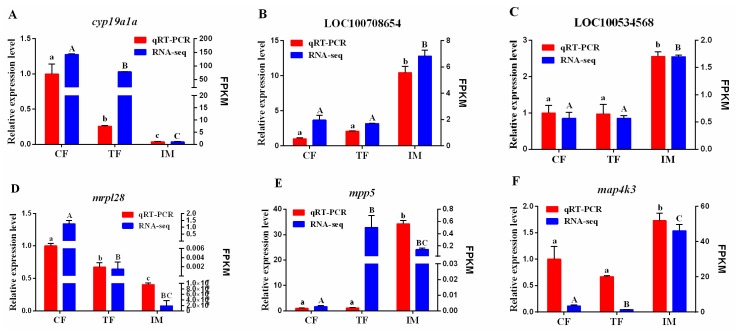
qRT-PCR validation of RNA-Seq data. (**A**): *cyp19a1a*; (**B**): LOC100708654; (**C**): LOC100534568; (**D**): *mrpl28*; (**E**): *mpp5*; (**F**): *map4k3*. The left *Y*-axis represent the relative expression level determined by qRT-PCR, and the right *Y*-axis represent FPKM determined by RNA-Seq. The scales of the left and right *Y*-axis are different. All data represent the mean value of three biological replicates. Error bars represent the standard errors of three replicates. Different lowercase letters (in qRT-PCR) and capital letters (RNA-seq) show statistical significance between variables, according to one-way ANOVA and post-hoc Duncan’s multiple range tests (*p* < 0.05).

To validate the transcriptome results, a total of six DEGs that showed significantly different expression levels in at least one comparison group was selected for qRT-PCR analysis. Among them, three DEGs encode enzymes participating in steroidogenesis, namely, *cyp19a1a*, LOC100708654 (17-β-hydroxysteroid dehydrogenase 7, *hsd17b7*), and LOC100534568 (3-β-hydroxysteroid dehydrogenase type I, *3β-hsd*), one DEG (*mrpl28*) was involved in energy metabolism and mitochondrial function, and two DEGs (*mpp5* and *map4k3*) were involved in ovary development. As shown in Figure 6, the expression patterns of the six DEGs identified by qRT-PCR were generally similar to those obtained in the RNA-Seq analyses, although the relative expression levels were not completely consistent. For *cyp19a1a* and *mrpl28*, RNA-Seq and qRT-PCR showed mRNA expression downregulation in TF and IM compared with CF (Figure 6A,D). However, for *mpp5*, RNA-Seq analyses showed upregulated expression in both TF and IM compared with CF, while qRT-PCR indicated that there were no significant differences between CF and TF (Figure 6E). qRT-PCR analysis further confirmed the reliability and accuracy of the RNA-Seq data.

## 3. Discussion

The molecular mechanism of TSD or GSD + TE in fish is still largely unknown. High throughput sequencing or microarray analysis of GSD + TE in zebrafish and European sea bass has been reported [15,31]. Global DNA methylation changes in Nile tilapia gonads during high-temperature-induced masculinization have also been analyzed [27]. In this study, an all-female Nile tilapia family was used for transcriptome analysis, and a series of high-temperature treatment-related genes were identified. Our study identified DEGs in the CF–TF, CF–IM, and CF–IM comparisons. Among the three groups, there were fewer DEGs in the CF–TF (79) than in the CF–IM (11,117) and TF–IM (11,000) comparisons, showing that the initiation of the male differentiation pathway after high-temperature treatment results in expression changes of many genes. The 44 shared DEGs having identical expression profiles in the CF–TF and CF–IM comparisons showed lasting effects of early exposure to high temperature on the gonadal transcriptome and important roles of these shared DEGs in Nile tilapia GSD + TE.

In this study, three all-female families were developed. The masculinizing effect of high temperature confirms previous results in Nile tilapia [10] and was similar to results in many other fish species reported in the literature [4,32]. Previously, it was reported that an influence of the paternal and maternal mating partners on the male proportions in temperature-treated progenies exists [9,27,33]. In this study, we also found that the male ratio after early high-temperature treatment displayed variation among but not within the three all-female families, suggesting that the Nile tilapia offspring sex ratio has a genetic component that is significantly influenced by the parental genotypes. Within each all-female family, not all offspring became sex-reversed males, and the resistance to high temperature resulted from individual differences. However, the development of all-female families made it easier to identify high temperature-induced neomales. Sequential gonad histology showed that ovaries developed earlier than testes. It was previously reported that meiosis became apparent in ovaries between 20 and 25 dph in Nile tilapia [34], and similar results were observed in this study. Kobayashi et al. suggested that spermatogenesis is not initiated until 70 dph in Nile tilapia [34]. Testes differentiation in the high temperature-induced group and the normal group did not occur until 55 dph. At 70 dph, germ cells of different types were found in normal male gonads. Interestingly, it seemed that the gonads of sex-reversed neomales developed slower than those of normal males and that most germ cells in the gonads remained at the spermatogonial stage at 70 dph. Although the high-temperature treatment delayed gonad development, gonads of IM matured, and milky milt could be squeezed out at six months. Combined with previous studies, our results suggest that high-temperature treatment causes a lag in spermatogenesis but does not affect the production of mature spermatozoa.

To exploit the effect of high-temperature treatment during early sex differentiation stages on gonad development, gonadal transcriptome analysis of Nile tilapia juveniles at 115 dph was performed. Since the CF, TF, and IM were from the same family, while the control males were from the other family, in order to use fish for transcriptome analysis with the same genetic background, only CF, TF, and IM were used for transcriptome analysis, and the control males were not included. We found that high-temperature-treated females (TF) had a gonadal transcriptome similar to that of control females (CF), with just a small number of DEGs. This observation was also reported for zebrafish [31], where only 20 DEGs were identified when comparing control females with high-temperature-treated females (FHT1). However, another type of high-temperature-treated female, which displayed a normal ovarian phenotype but a “male-like” gonadal transcriptome (FHT2), was found in zebrafish [31]. FHT2-type fish were not observed in the current study. In this study, the sex-reversed males (IM) had a gonad transcriptome that was quite different from that of CF and TF, i.e., it differed by 11,117 and 11,000 DEGs from CF and TF, respectively. According to these results, we can speculate that the gonad transcriptome of Nile tilapia juveniles after high-temperature treatment at 5–17 dph is significantly altered, and that more than 79 DEGs, as found in the CF–TF comparison in this study, will be identified. In red-eared slider turtles (*Trachemys scripta*), more than 500 annotated genes are significantly differentially expressed in stage-12 embryos (prior to gonad formation) incubated at MPT (male-producing temperature) versus FPT (female-producing temperature) [35]. Previously, many sex differentiation-related genes involved in masculinization after high-temperature treatment were found in fish, usually a result of downregulation of female-related genes, such as *cyp19a1a* and *foxl2*, and upregulation of male-related genes, such as *dmrt1* or *amh* [15,16,19,20,21]. Therefore, we further speculate that the high-temperature treatment of Nile tilapia juveniles at 5–17 dph will result in the inhibition of some female sex differentiation-related genes immediately after high-temperature treatment and in the activation of many male sex differentiation-related genes later. It is very necessary that an analysis of the gonadal transcriptome changes of Nile tilapia juveniles after high-temperature treatment at 5–17 dph is conducted in the future.

In this study, one important finding was that among the 79 DEGs identified in the CF–TF comparison, 54 DEGs were shared with the DEGs found in the CF–IM comparison. Furthermore, most of them (44 DEGs, accounting for 81.5% of the total shared DEGs) showed identical expression changes in the two comparisons. These 44 DEGs were found in many sex differentiation-related KEGG pathways, such as steroid hormone biosynthesis (ko00140), and apperead as important candidate genes for investigating the molecular mechanisms for fish TSD or GSD + TE. Additionally, we identified many steroidogenesis-related genes, which play an important role in sex differentiation [25], in the CF–TF or CF–IM comparisons. Androgen biosynthesis-related genes, such as *3β-hsd*, *hsd17b8*, and *cyp1b1* were significantly upregulated in IM compared with CF, but, interestingly, not in TF. In red-eared slider turtles, it was found that the expression of Akr1d1 (5β-reductase) was increased at MPT, which reduced the pool of testosterone that could be aromatized to form estrogens [35]. In this study, *cyp19a1a*, which is responsible for the production of 17β-estradiol [25] and a key gene responsible for sex differentiation, was significantly downregulated in both IM and TF compared with CF. Downregulation of *cyp19a1a* highlighted the lasting effect of high-temperature treatment during early sex differentiation stages in Nile tilapia. High-temperature treatment during European sea bass early developmental stages increased DNA methylation of the cyp19a1a promoter, with an observed inverse relationship between methylation levels and expression, resulting in male-biased sex ratio shifts [16]. Our previous study also showed that high-temperature treatment during early sex differentiation stages increased *cyp19a1a* promoter DNA methylation levels in four-month-old Nile tilapia [36]. Collectively, DNA methylation-mediated mRNA expression changes of many important sex differentiation-related genes during temperature treatment possibly play an important role in fish TSD or GSD + TE [16,37].

Nile tilapia has a wide thermal tolerance zone, and the critical thermal maximum was 40.3–40.5 °C [38]. The treatment temperature used in this study, 36 °C, was below the critical thermal maximum in Nile tilapia. Since the Nile tilapia neomales obtained by artificial high-temperature treatment were fertile in this study, we question if wild Nile tilapia populations exposed to abnormally high temperature will produce a proportion of sex-reversed neomales. Furthermore, the presence of neomales has been strongly suggested in Kpandu and Koka natural populations [39]. If they reach adulthood and reproduce, neomales should produce 100% females, considering that Nile tilapia has a primarily XX/XY sex determining system. Currently, TSD or GSD + TE has been found in many fish species, such as *Atlantic silverside* [5], zebrafish [31], tongue sole [40], Japanese flounder [41], Nile tilapia [36], etc. Furthermore, it is found that methylation modifications in tongue sole pseudomales are globally inherited in their ZW offspring, which can naturally develop into pseudomales without temperature treatment [40]. In this study, the expression of 79 DEGs in high-temperature-treated females was changed. In zebrafish, a female with a male-like transcriptome was observed [31]. In the future, understanding the reproduction of these females would be a challenge for endocrinologists and reproductive physiologists alike [31]. 

## 4. Materials and Methods

### 4.1. Fish Culture and Family Development

Sixteen three-year-old Nile tilapia, 12 females and 4 sex-reversed neomales, were collected from the Guangxi Fisheries Institute (Nanning, China) and reared in 100 m^3^ tanks in the experimental base of the Guangxi Fisheries Institute (Nanning, China). These Nile tilapia were introduced into Guangxi Fisheries Institute from Qingdao National Nile tilapia seed farm (Qingdao, China) in 1994. The mixed sex larvae at sex differentiation stage were fed diets with 17α-methyltestosterone (MT) (obtained by dissolving 20 mg of 17α-MT in one liter of 95% ethanol and then spraying it on one kilogram of diet) according to a previous report for sex reversal [42]. The neomales were identified from the methyltestosterone-treated group using the screened sex-linked DNA markers (SCAR-5F and SCAR-5R) suggested by Sun et al. [43] (Table S6), and the neomales could produce semen by applying a gentle abdominal pressure. The fish were fed pelleted Nile tilapia food of appropriate size once daily. After two weeks, the 16 tilapia were divided into four groups. Each group contained three females and one sex-reversed neomale cultured in a 20 m^3^ tank under a natural photoperiod and water temperature (23–29 °C). The fishes’ mouths were checked every five days. Any embryos in the mouth were taken out and cultivated artificially in a 0.5 m^3^ aquarium per family, independently. There was one aerating stone in each 0.5 m^3^ aquarium, and the water temperature was controlled at 28–29 °C. In total, three all-female families were developed in this study for high-temperature treatment, as three biological replicates.

Normal females were crossed with normal males in Guangxi Fisheries Institute, and the obtained larvae were cultured under a natural photoperiod and water temperature (23–29 °C). At 20, 30, 45, 60, and 75 dph, the male fish were sampled for histological analysis.

### 4.2. High-Temperature-Induced Masculinization

The high-temperature-induced masculinization of Nile tilapia was performed as previously described [9,27]. Briefly, at five days post-hatching (dph), approximately 500 larvae from each family were equally divided into two groups and were cultured in two 50 L tanks. One group served as the control, and the larvae were cultivated at 28 °C. The other was the high-temperature-treated group, and the larvae were cultured at 36 °C for 12 days, which promoted male development. Thereafter, the larvae of the high-temperature-treated group were gradually adapted to 28 °C again. The larvae from the control and high-temperature-treated groups were cultured at a natural water temperature (21–30 °C) until sampling. The fish were fed pelleted tilapia food of the appropriate size two to three times daily.

### 4.3. Sampling

The study was approved by Shandong Agricultural University Animal Care and Use Committee with approval number SDAUA-2015-017 on 12 May 2015. In all cases, the fish were treated in agreement with the Shandong Agricultural University Animal Care and Use Committee. At 20, 30, 45, 60, 75, and 115 dph, the fish were individually anaesthetized with 0.2% MS-222 and then sacrificed. The main body of larvae at 20 and 30 dph and the gonads of juveniles at 45, 60, and 75 dph were immersed in Bouin’s solution for histological analysis. For 115 dph fish, one gonad was processed for histological sex identification, and the other gonad was frozen in liquid nitrogen for RNA extraction. The percentage of males in the high-temperature-treated group of each family was determined according to the histological analysis results. The gonads of three females in the control group (CF), three females in the high-temperature-treated group (TF), and three neomales in the high-temperature-induced group (IM) in family 3 were used for RNA-Seq and qRT-PCR analysis.

### 4.4. Histological Analysis

The fixed samples were dehydrated and embedded in paraffin; 5-μm-thick (paraffin) sections were cut, and the paraffin sections were stained with hematoxylin–eosin for histological examination with a light microscope [27].

### 4.5. Illumina Sequencing and Quality Control

Total RNA was isolated from each tissue sample using Trizol reagent (Takara, Shiga, Japan) according to the manufacturer’s instructions. The quantity and quality of the RNA samples were determined using a Nanodrop 2000 (Thermo Fisher Scientific, Wilmington, DE, USA) and an Agilent 2100 Bioanalyzer (Agilent Technologies, Singapore). Samples with RNA integrated quality (RIQ) numbers ranged from 7.1 to 9.9. Total RNA (~5 μg) was used for library preparation and sequencing. The libraries were developed using the Illumina HiSeq RNA library method according to the TrueSeq RNA Sample Preparation guide (Illumina Technologies, San Diego, CA, USA), and paired-end sequencing was done using Illumina Genome Analyzer II (Illumina) to obtain reads of approximately 100 bp in length. The sequencing reads were filtered using NGSQC Toolkit28 at default parameters for removing low-quality (Q < 30) and primer/adapter-contaminated reads. The proportion of reads with an average Q value ≥ 30 was 96.36%, suggesting a high quality of the sequencing data and suitability for further analysis. All datasets from the Illumina sequencing platform can be found in the Short Read Archive (SRA) database of the National Center for Biotechnology Information (NCBI) under accession number PRJNA387639.

### 4.6. Reference-Based Assembly

TopHat and Cufflinks software were used for reference-based assembly [44]. TopHat was used for mapping the HQ reads on the Nile tilapia genome, version Orenil1.1 (https://www.ncbi.nlm.nih.gov/assembly/GCF_000188235.2/). The assembly was performed via Cufflinks using the TopHat mapping files with default parameters. The final assembly was obtained by merging the individual assemblies with default options using Cuffmerge. Functional annotation of the assembled transcripts was carried out using blastx search with an *E*-value cut-off of ≤1 × 10^−5^ against the Nile tilapia proteome sequence and non-redundant (NR) protein database from NCBI. The novel transcripts/transcript isoforms were identified using Cuffcompare with default parameters. The annotation of novel genes/transcripts was carried out using blastx search against the NR database. We filtered the output using an E-value cut-off ≤ 1 × 10^−5^ to assign a putative function to the transcripts.

### 4.7. Differential Gene Expression

Differential gene expression was determined by the Cuffdiff utility provided in the Cufflinks package. Transcripts with a fold change ≥ 1.5 and false discovery rate (FDR) ≤ 0.05 were considered to be significantly differentially expressed transcripts.

### 4.8. Gene Ontology Enrichment and Pathway Analysis

Gene ontology enrichment for various differentially expressed transcripts was performed using the BiNGO plug-in of the Cytoscape platform. Nile tilapia GO information for biological processes and molecular functions was used for gene ontology enrichment analysis. We considered a *p*-value cut-off ≤ 0.05 as significant and applied the hypergeometric test to identify enriched GO terms in BiNGO. The pathway analysis of differentially expressed transcripts was carried out using Mapman with a *p*-value ≤ 0.05. The differentially expressed transcripts were mapped on Nile tilapia pathway genes to identify the transcripts involved in specific pathways.

### 4.9. Quantitative PCR Analysis

The validation of RNA-Seq data was carried out using qRT-PCR analysis. The RNA used for qPCR was the same as the one used for RNAseq. Primers for each gene were designed using Primer Premier 6.0 (PREMIER Biosoft International, Palo Alto, CA, USA), and the primer sequences are shown in Table S6. Quantitative real-time PCR (RT-qPCR) was conducted in an Mx3000p™ real-time PCR system (Agilent Technologies, Santa Clara, CA, USA). The RT-qPCR reaction system (20 μL) comprised 10 μL of SYBR Premix Ex Taq (2×) (Takara, Japan), 0.4 μL of each gene-specific primer (10 nmol), 2 μL of cDNA, and 0.4 μL of ROX reference dye II. The PCR amplification procedure comprised an initial denaturation step at 95 °C for 30 s, 40 cycles at 95 °C for 5 s, 60 °C for 30 s, and 72 °C for 30 s, followed by disassociation curve analysis to determine target specificity. The expression of β-actin was used as an internal control [7]. Three replicates for each gene and for each individual were performed, and the fluorescence intensities of each gene, as measured by cycle threshold (*C*t) values, were compared by the 2(−∆∆*C*t) method [45]. PCR specificity was assessed by melting curve analysis.

### 4.10. Statistical Analysis

All data are expressed as means ± S.E (standard error). One-way ANOVA and post-hoc Duncan’s multiple range tests were used to determine the difference between groups using SPSS 11.0. Differences were considered to be statistically significant at *p* < 0.05.

## 5. Conclusions

All-female families were used for analyzing the gonadal gene expression profile after high-temperature treatment during early sex differentiation stages in Nile tilapia. The results of this study indicated that high-temperature-treated females had similar gonadal expression profiles as control females. The gonad transcriptome profile of IM was different from those of CF or TF. Many DEGs, involved in some sex differentiation-related KEGG pathways, indicated identical expression changes in the CF–TF and CF–IM comparisons. These DEGs appeared as important candidate genes for investigating the molecular mechanisms for fish TSD or GSD + TE. These findings pave the way to investigate the mechanism underlying high-temperature-induced masculinization in Nile tilapia.

## Figures and Tables

**Table 1:** Number of differentially expressed genes (DEGs).

Comparisons	Upregulated	Downregulated	Total
CF–TF	34	45	79
CF–IM	5779	5338	11,117
TF–IM	5607	5393	11,000

**Table 2:** Forty-five DEGs shared by the CF–TF and CF–IM comparisons.

GenBank No.	Gene Name	Up or Down	
		CF/TF	CF/IM
AF135851_1	Cytochrome P450 aromatase (*cyp19a1a*)	Down	Down
XP_005467510.1	Guanine nucleotide exchange factor DBS-like	Down	Down
XP_003447876.2	Syncoilin-like isoform X1	Down	Down
XP_005478344.1	Dihydroxyacetone phosphate acyltransferase-like	Down	Down
XP_003439429.1	Peptide deformylase, mitochondrial-like	Down	Down
XP_003456103.1	Protein kish-A-like	Down	Down
XP_005467797.1	Protocadherin-10-like	Down	Down
XP_003445105.1	MOB kinase activator 3A-like	Down	Down
XP_005452095.1	Netrin receptor UNC5C-like isoform X1	Down	Down
XP_003455736.2	Zinc transporter 6-like isoform X1	Down	Down
XP_005456672.1	Free fatty acid receptor 4-like	Down	Down
XP_003450852.1	39S ribosomal protein L28, mitochondrial-like	Down	Down
XP_003448430.2	1-phosphatidylinositol 4,5-bisphosphate phosphodiesterase eta-2-like	Down	Down
XP_005459015.1	Excitatory amino acid transporter 3-like isoform x1	Down	Down
XP_005457662.1	Fmet-leu-phe receptor-like	Down	Down
XP_005946247.1	Uncharacterized protein loc102297687 isoform x1	Down	Down
XP_003456623.1	Ganglioside-induced differentiation-associated protein 2-like isoform x1	Down	Down
XP_005476479.1	Arf-gap with gtpase, ank repeat, and ph domain-containing protein 3 isoform x2	Down	Down
XP_005477055.1	Atp-dependent rna helicase *ddx24*	Down	Down
XP_005452886.1	Integrator complex subunit 8 isoform x2	Down	Down
XP_003452492.1	Ictacalcin-like	Down	Down
XP_003440093.1	Lipid phosphate phosphohydrolase 2-like isoform x1	Down	Down
XP_005467551.1	Forkhead box protein O4-like isoform x1	Up	Up
XP_005456696.1	Acyl-coa synthetase short-chain family member 3	Up	Up
XP_003437928.1	Atpase wrnip1-like isoform x1	Up	Up
XP_005464160.1	Uncharacterized protein loc102077664	Up	Up
XP_005464413.1	Butyrophilin-like protein 10-like	Up	Up
XP_005460191.1	Fibrinogen c domain-containing protein 1-like	Up	Down
XP_003446337.1	Maguk p55 subfamily member 5-a-like	Up	Up
XP_005802162.1	Uncharacterized protein loc102218015	Up	Up
XP_006788818.1	Gpn-loop gtpase 3-like	Up	Up
XP_009302985.1	Uncharacterized protein loc103911649	Up	Up
XP_005449253.1	Uncharacterized protein loc100704487 isoform x2	Up	Up
XP_005465760.1	Up-regulator of cell proliferation-like	Up	Up
XP_003442517.2	Uncharacterized protein loc100698127	Up	Up
XP_005953198.1	Caspase-1-like	Up	Up
XP_003452737.1	Cell division control protein 42 homolog isoform x1	Up	Up
XP_004553491.1	Mapk4-like isoform x5	Down	Up
XP_003454726.1	Fas apoptotic inhibitory molecule 1-like	Down	Down
XP_005458342.1	Pyrroline-5-carboxylate reductase 3-like isoform x2	Down	Up
XP_003454060.1	Transmembrane protein 11	Down	Up
XP_003444492.1	Sigma non-opioid intracellular receptor 1-like	Down	Up
XP_005470690.1	Fatty acid desaturase 2-like isoform x1	Down	Up
XP_005464685.1	Breast cancer anti-estrogen resistance protein 1-like	Up	Down
XP_005476032.1	α-2-hs-glycoprotein-like	Up	Down

## Supplementary Materials

Supplementary materials can be found at http://www.mdpi.com/1422-0067/19/3/689/s1.
