# Supplementary material for: Gonad Transcriptome Analysis of High-Temperature-Treated Females and High-Temperature-Induced Sex-Reversed Neomales in Nile Tilapia
Source: Int J Mol Sci. 2018 Feb 28;19(3):689. doi: 10.3390/ijms19030689 (PMC5877550; doi:10.3390/ijms19030689)
Supplement: Supplementary file 1 [file ijms-19-00689-s001.zip › Supplementary/Supplmentary figures .docx]

Article

Gonad Transcriptome Analysis of High-Temperature-Treated Females and High-Temperature-Induced Sex-Reversed Neomales in Nile Tilapia


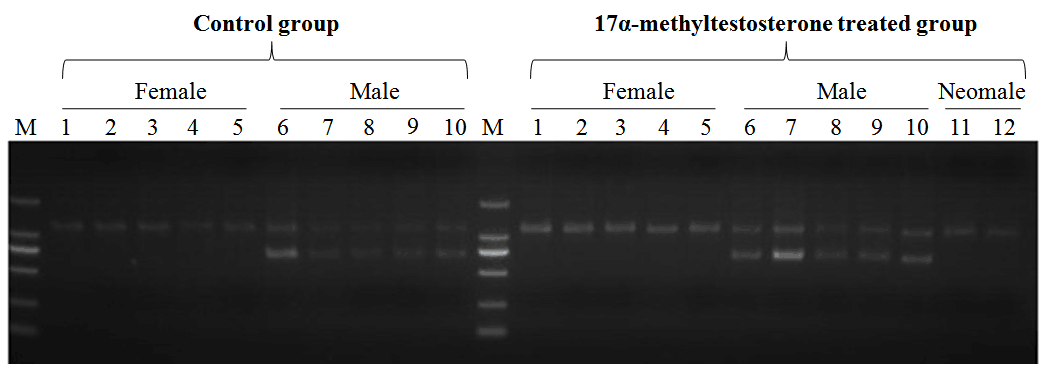


**Figure S1.** Genetic sex identiﬁcation of the Nile tilapia from control and 17α-methyltestosterone (MT) treated groups by SCAR markers. Genetic females (with XX chromosomes) are identified by a single X-specific band, and genetic males (with XY chromosomes) iares identified by two X- and Y-specific bands. An amount of 20 mg of 17α-MT was dissolved in one litre of 95% ethanol and then sprayed on one kilogram of diet. Neomale: genetically female but physiologically male (with a single band). M: DL2000 marker.


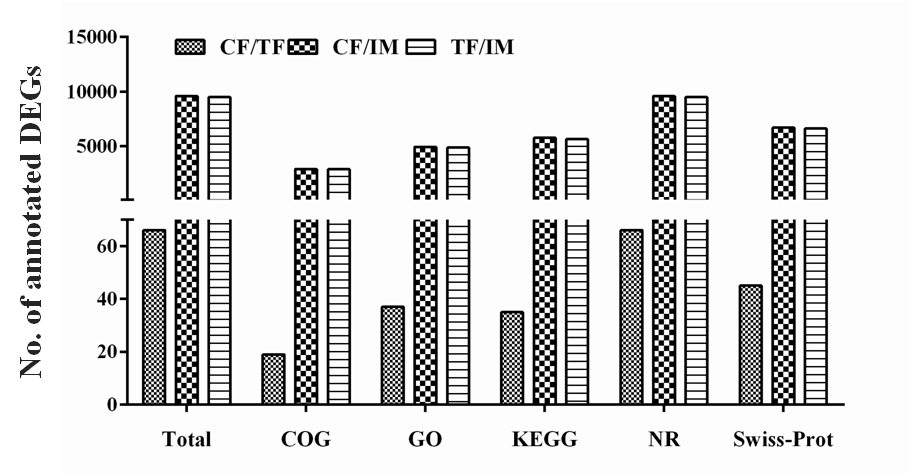


**Figure S2.** Numbers of annotated DEGs from the three comparison groups against different databases.

**
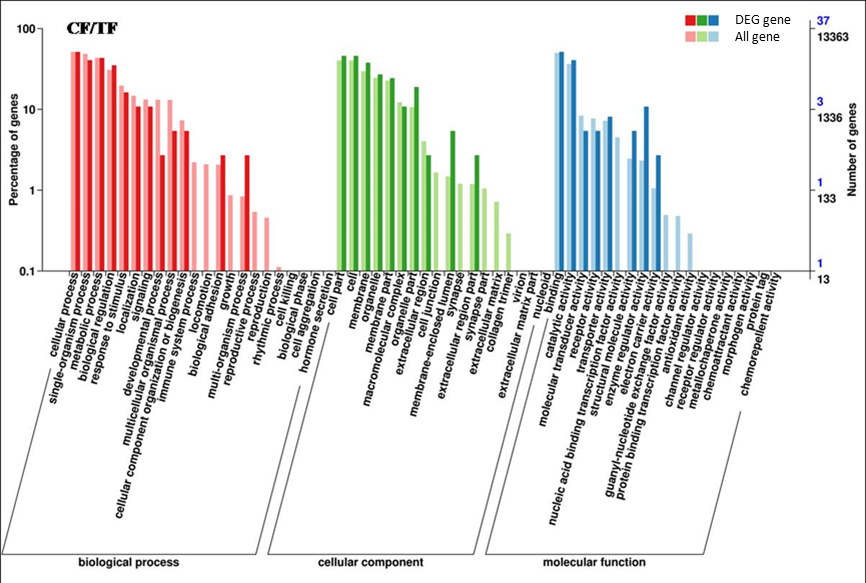
**

**Figure S3.** Gene ontology classification of the Nile tilapia gonad transcriptome data in the CF–TF comparison. The unigenes were classified at the second level under three GO domains: cellular component (CC), molecular function (MF), and biological process (BP). The right and left y-axis represent the number of genes and the corresponding percentage of genes in various ontologies, respectively. One gene can be annotated into more than one GO term.

**
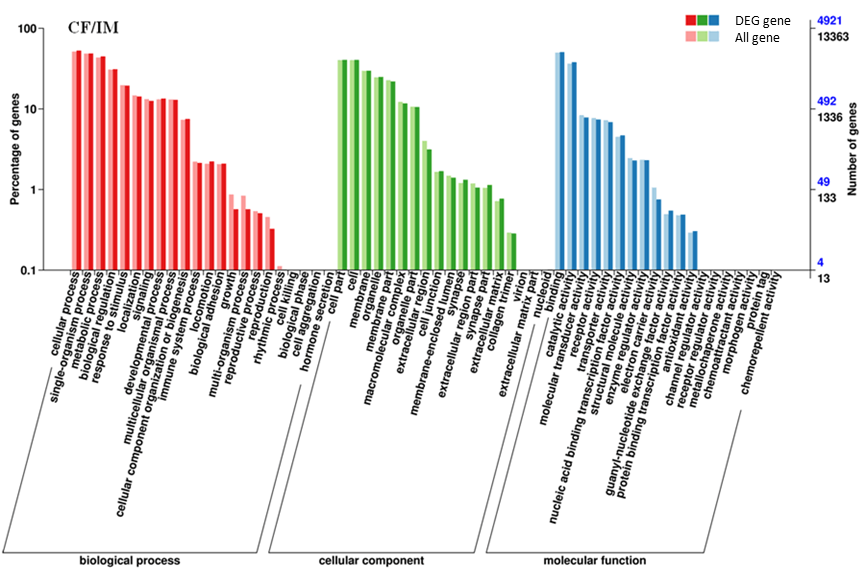
**

**Figure S4.** Gene ontology classification of the Nile tilapia gonad transcriptome data in the CF–IM comparison. The unigenes were classified at the second level under three GO domains: cellular component (CC), molecular function (MF), and biological process (BP). The right and left y-axis represent the number of genes and the corresponding percentage of genes in various ontologies, respectively. One gene could be annotated into more than one GO term.

**
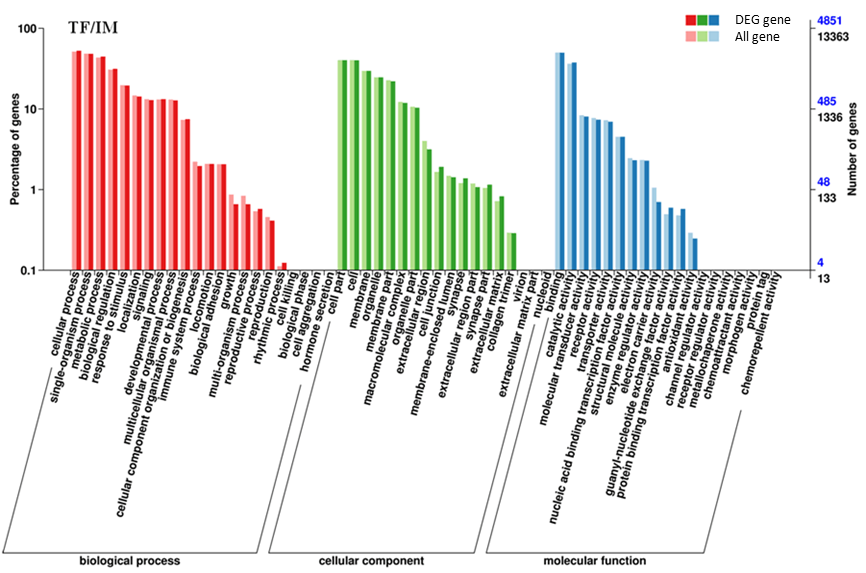
**

**Figure S5.** Gene ontology classification of the Nile tilapia gonad transcriptome data in the TF–IM comparison. The unigenes were classified at the second level under three GO domains: cellular component (CC), molecular function (MF), and biological process (BP). The right and left y-axis represent the number of genes and the corresponding percentage of genes in various ontologies, respectively. One gene could be annotated into more than one GO term.
